# Point of Care eGFR and the Prediction of Outcomes in Pneumonia

**DOI:** 10.1038/s41598-019-44945-2

**Published:** 2019-06-11

**Authors:** Chi-won Suk, Shih-chang Hsu, Chun-you Chen, Hui-ling Hsieh, Hsiao-tung Kuo, Yuan-pin Hsu, Yuh-mou Sue, Tso-Hsiao Chen, Feng-yen Lin, Chun-ming Shih, Jaw-wen Chen, Shing-jong Lin, Po-hsun Huang, Chung-te Liu

**Affiliations:** 1 Division of Pulmonary Medicine, Department of Internal Medicine, Wan Fang Hospital, Taipei Medical University, Taipei, Taiwan; 2 Emergency Department, Department of Emergency and Critical Medicine, Wan Fang Hospital, Taipei Medical University, Taipei, Taiwan; 3 Department of Emergency Medicine, School of Medicine, College of Medicine, Taipei Medical University, Taipei, Taiwan; 4 Department of Radiation Oncology, Wan Fang Hospital, Taipei Medical University, Taipei, Taiwan; 5 Division of Nephrology, Department of Internal Medicine, Wan Fang Hospital, Taipei Medical University, Taipei, Taiwan; 6 Graduate Institute of Medical Science, National Defense Medical Center, Taipei, Taiwan; 7 Graduate Institute of Clinical Medicine, College of Medicine, Taipei Medical University, Taipei, Taiwan; 8 Department of Internal Medicine, School of Medicine, College of Medicine, Taipei Medical University, Taipei, Taiwan; 9 Division of Cardiology and Cardiovascular Research Center, Department of Internal Medicine, Taipei Medical University Hospital, Taipei, Taiwan; 10 Division of Cardiology, Department of Medicine, Taipei Veterans General Hospital, Taipei, Taiwan; 11 Institute of Clinical Medicine, National Yang-Ming University, Taipei, Taiwan; 12 Cardiovascular Research Center, National Yang-Ming University, Taipei, Taiwan; 13 Department of Medical Research, Taipei Veterans General Hospital, Taipei, Taiwan; 14 Institute of Pharmacology, National Yang-Ming University, Taipei, Taiwan; 15 Board of Directors, Taipei Medical University, Taipei, Taiwan

**Keywords:** Acute kidney injury, Chronic kidney disease

## Abstract

Pneumonia is a leading cause of mortality. Severity-assessment scores in pneumonia guide treatment crucially, but the ones currently in existence are limited in their use. Community-based studies demonstrated the association between pre-existing low estimated glomerular filtration rate (eGFR) and outcomes in pneumonia. However, whether a single emergency department-eGFR measurement could predict outcomes in pneumonia remains unclear. This retrospective cohort study included 1554 patients hospitalized with pneumonia. The predictor was the first eGFR measurement. Outcomes included mortality, intensive care unit (ICU) admission, durations of hospital and ICU stay, and ventilator use. Receiver operating characteristic curves was used to determine optimal cutoff values to predict mortality. Of 1554 patients, 263 had chronic kidney disease, demonstrated higher C-reactive protein and SMART-COP scores, and had more multilobar pneumonia, acute kidney injury, ICU admission, and mortality. Patients with higher pneumonia severity scores tended to have lower eGFR. For predicting in-hospital mortality, the optimal eGFR cutoff value was 56 mL/min/1.73 m^2^. eGFR < 56 mL/min/1.73 m^2^ had an odds ratio of 2.5 (95% confidence interval, 1.6–4.0) for mortality by multivariate logistic regression. In Conclusion, eGFR < 56 mL/min/1.73 m^2^ is an independent predictor of mortality, indicating that even mild renal impairment affects the outcome of pneumonia adversely.

## Introduction

Pneumonia is one of the leading causes of mortality globally^1–3^. Despite the advancements in medical care, the 30-day mortality of community-acquired pneumonia remains as high as 12.1% in elderly patients^4^. In the emergency department, severity stratification is essential for the treatment of patients with pneumonia^5–8^ as early determination of the severity of pneumonia improves its outcomes^9,10^. Hence, several severity-assessment indices for pneumonia have been developed, such as the Pneumonia Severity Index (PSI)^9,11,12^, CURB-65 score^11,13^, and SMART-COP score^14^. However, the complexities of PSI and SMART-COP score and the underestimation of severity in young patients by PSI and CURB-65 limit the application of these scoring systems^15^. Additionally, these scoring systems are considered suboptimal in predicting the need for intensive care unit (ICU) admission^15,16^. Therefore, a simpler and more comprehensive severity-assessment index is desired.

Improving the outcomes of pneumonia in patients with chronic kidney disease (CKD) is an important issue^17^. Patients with CKD are at increased risk of infection-related hospitalization. Infection ranked as the second leading cause of mortality in patients with end-stage renal disease (ESRD)^18^, and pneumonia is one of the most common infectious diseases in patients with CKD^19–21^. Moreover, renal impairment is associated with adverse outcomes in pneumonia. Several community-based studies demonstrated that CKD is associated with an increased risk of pneumonia-associated hospitalization and mortality^22–24^. Furthermore, acute kidney injury (AKI) is associated with adverse outcomes in patients hospitalized with pneumonia^25,26^. Thus, previous studies indicated that both acute and chronic renal impairments are associated with adverse outcomes in pneumonia. However, the capability of eGFR as a predictor of outcomes in pneumonia remains to be investigated.

Decreased eGFR may be a potential predictor of adverse outcomes in patients with pneumonia. Nonetheless, owing to the broad spectrum of the manifestations of renal diseases, ranging from asymptomatic azotemia to uremic symptoms, an optimal cutoff eGFR value to predict pneumonia-related mortality remains to be determined. Therefore, we conducted a retrospective cohort study to identify the cutoff eGFR value to predict the mortality in patients hospitalized with pneumonia.

## Materials and Methods

### Study participants and study design

Patients hospitalized with pneumonia at Wan Fang Hospital, Taipei Medical University between January 2013 and December 2015 were enrolled in eligibility review. The inclusion criteria included patients admitted via the emergency department, and the primary diagnosis at discharge was community-acquired or healthcare-associated pneumonia. The exclusion criteria were age <20 years old. The medical records of eligible patients were reviewed from January 1, 2018 to March 30, 2018. After the eligibility review, 1554 of the 1831 reviewed patients were included into the study. This study was approved by the ethics committee and Institutional Review Board of Taipei Medical University (N201805061) and was conducted in accordance with the tenets of the 1975 Declaration of Helsinki, as revised in 2000. The present study did not involve clinical trial or experiment of tissue samples and informed consent was waived according to the policy of Institutional Review Board.

### Measurement of covariates and definition of outcomes

Baseline laboratory data and physical measurements, which were obtained on the day of the visit to the emergency department, were used. CKD was defined as serum creatinine level >1.3 mg/dL for >3 months before the index hospitalization. AKI was defined as serum creatinine level elevation by ≧0.5 mg/dL from the latest value before the admission. For patients without documented past creatinine levels, CKD and AKI were defined based on the discharge diagnosis. ESRD was defined as initiation of maintenance dialysis before the indexed hospitalization. For patients with ESRD, AKI was considered absent irrespective of the change in serum creatinine levels. Other comorbidities were defined according to the International Classification of Disease, 10th Revision, clinical modification codes of the discharge diagnoses. Severity of pneumonia was quantified based on CURB-65^13^ and SMART-COP scores^14^, and the risk stratification groups were according to the original publications. In this study, eGFR was calculated using the equation suggested by the Chronic Kidney Disease Epidemiology Collaboration in 2009, in which serum creatinine level, age, and gender were required for calculation of eGFR^27^. Notably, the eGFR of patients with ESRD was uniformly regarded as 5 mL/min/m^2^ in the statistical analyses for two reasons: First, eGFR calculated from the serum creatinine levels that fluctuate with hemodialysis is not applicable for renal function evaluation. Second, eGFR <5 mL/min/m^2^ is the cutoff value for the initiation of maintenance dialysis according to the National Health Insurance of Taiwan.

For evaluation of the pneumonia severity score, SMART-COP and CURB65 were used. SMART-COP was designed to predict the need for intensive respiratory or vasopressor support in pneumonia patients, which includes low blood pressure, multilobar pneumonia, low serum albumin level, tachypnea, tachycardia, hypoxemia, and acidosis. Notably, acidosis was defined as arterial pH <7.35 regardless metabolic or respiratory origin. CURB65, which was design to predict the mortality of pneumonia patients, includes conscious confusion, elevated BUN level, tachypnea, hypotension and age >65 years as its parameters.

The primary outcome in this study was in-hospital mortality, which was defined as mortality at the end of the indexed hospitalization. The secondary outcomes included the duration of hospitalization, admission to the ICU, duration of ICU care, and the use of ventilators and its duration.

### Statistics

In this study, continuous variables with normal distribution were reported as mean ± standard deviation, while continuous variables without normal distribution were expressed as median (25th and 75th percentiles). Categorical variables were reported as frequency and percentage. Comparisons of continuous variables were made using a two-tailed t-test for unpaired samples or non-parametric methods, as appropriate; comparisons of categorical variables, using the chi-squared test. Receiver operating characteristic (ROC) curve with Youden criterion was employed to determine the optimal cutoff eGFR value for predicting in-hospital mortality. To investigate the association between predictors and in-hospital mortality, the predictors with p values ≦ 0.2 in the univariate logistic regression were included in the multivariable logistic regression model. The association between predictors and outcomes in the logistic regression model was expressed as odds ratio (OR) and 95% confidence interval (CI). Statistical analysis was performed using SAS 9.4 (SAS Institute Inc., Cary, NC, USA). Statistical power was calculated using G*Power 3.1.9.2 (Franz Faul, Universität Kiel, Germany) and confidence interval was calculated using calculator provided at: https://www.surveysystem.com/sscalc.htm#one.

## Results

### Demographic and laboratory characteristics

**Figure 1:**
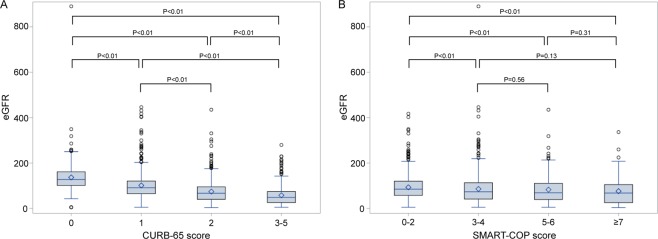
eGFR values of groups stratified by pneumonia severity scores. (**A**) eGFR values of groups stratified by CURB-65 score. (**B**) eGFR values groups stratified by SMART-COP score. eGFR, estimated glomerular filtration rate. The difference between the groups were compared by Exact Wilcoxon two-sample test.

This study included 1554 patients who were hospitalized with pneumonia. The mean age was 76.1 ± 16.7 years. Of the enrolled patients, 987 (63.5%) were male, and 263 (16.9%) had CKD. Compared with patients without CKD, those with CKD were of older age, included more males and those with diabetes, had lower hemoglobin levels, and higher blood urea nitrogen, serum creatinine, C-reactive protein (CRP), and potassium levels. The eGFR of those with and without CKD was 25.7 and 89.4 mL/min/1.73 m^2^, respectively (Table 1). Upon stratification by CURB-65 score (grouped according to 0, 1, 2, and 3–5 points), eGFR was significantly lower in groups with higher severity (Fig. 1A). Similarly, on stratification by SMART-COP score (grouped according to 0–2, 3–4, 5–6, and ≧7 points), the groups with severity scores of 3–4, 5–6, and ≧7 showed significantly lower eGFR than the group with scores of 0–2. However, the difference in eGFR between the groups with severity scores of 3–4, 5–6, and ≧7 was not significant (Fig. 1B). These findings suggest decreased renal function in patients with higher pneumonia severity.

**Table 1:** Baseline demographics and laboratory characteristics.

Characteristics	Total	Non-CKD	CKD	p value
Number	1554	1291	263	N/A
Age	76.1 ± 16.7	75.1 ± 17.3	81.1 ± 12.1	<0.01
Male	987 (63.5%)	781 (60.5%)	206 (78.3%)	<0.01
CAD	60 (3.9%)	45 (3.5%)	15 (5.7%)	0.09
COPD	102 (6.6%)	90 (7.0%)	12 (4.6%)	0.15
DM	110 (7.1%)	80 (6.2%)	30 (11.4%)	<0.01
Hb (g/dL)	11.8 ± 2.2	12.1 ± 2.1	10.4 ± 2.1	<0.01
Albumin (g/dL)	2.9 ± 0.6	2.9 ± 0.6	2.9 ± 0.6	0.21
BUN (mg/dL)	20 (13, 32)	18 (12, 26)	42 (27, 61)	<0.01^a^
Creatinine (mg/dL)	0.9 (0.7, 1.4)	0.9 (0.7, 1.1)	2.2 (1.6, 4.4)	<0.01^a^
eGFR (mL/min/1.73 m^2^)	78.9 (48.6, 115.0)	89.4 (63.5, 124.1)	25.7 (5.0, 40.4)	<0.01^a^
CRP (mg/dL)	7.6 (2.8, 14.4)	7.3 (2.5, 13.8)	9.3 (4.5, 16.7)	<0.01^a^
AST (U/mL)	25 (19, 35)	25 (19, 34)	26 (19, 36)	0.17^a^
ALT (U/mL)	19 (13, 29)	19 (13, 29)	17.5 (12, 27)	0.11^a^
Na (mmol/L)	134.2 ± 7.1	134.0 ± 7.1	135.0 ± 7.0	0.04
K (mmol/L)	4.1 ± 0.7	4.0 ± 0.6	4.3 ± 0.8	<0.01

CKD, chronic kidney disease; CAD, coronary artery disease; COPD, chronic obstructive pulmonary disease; DM, diabetes mellitus; Hb, hemoglobin; BUN, blood urea nitrogen; eGFR, estimated glomerular filtration rate; CRP, C-reactive protein; AST, aspartate transaminase; ALT, alanine transaminase.

^a^Compared by Exact Wilcoxon two-sample test.

### CKD and outcomes of pneumonia

Of the 1554 included patients, there were 162 (10.4%) in-hospital deaths; the median hospital duration was 11 days (25^th^ and 75^th^ quartile: 7 and 18 days); 238 (15.3%) patients were admitted to ICU and 271 (17.4%) used invasive or non-invasive ventilators. In addition, 277 (17.8%) patients had AKI on admission to emergency department and 162 (10.4%) mortality were noted at the end of hospitalization. Systolic blood pressure and events of fever, tachypnea, tachycardia, acute confusion, hypoxemia, and acidosis were not significantly different between the patients with and those without CKD. Patients with CKD had pneumonia with more multilobar involvement based on chest x-ray, higher SMART-COP score, and more AKI events. Moreover, they also had significantly higher ICU admission (non-CKD versus CKD: 19.8% versus 14.4%) and in-hospital mortality rates (non-CKD versus CKD: 9.3 versus 16.0%) and longer hospitalization (median hospital stay of non-CKD versus CKD: 10 versus 12 days) in patients with CKD. Ventilator support was not significantly different between those with and those without CKD (Table 2).

**Table 2:** Severity of pneumonia of the patients admitted.

Characteristics	Total	Non-CKD	CKD	p value
Triage				N/A
1	381 (24.5%)	302 (23.4%)	79 (30.0%)	
2	466 (30.0%)	382 (29.6%)	84 (31.9%)	
3	693 (44.6%)	596 (46.2%)	97 (36.9%)	
4	13 (0.8%)	10 (0.8%)	3 (1.1%)	
5	1 (0.1%)	1 (0.1%)	0 (0%)	
Fever	156 (10.0%)	138 (10.7%)	18 (6.8%)	0.06
SBP (mmHg)	131.8 ± 24.0	131.9 ± 23.2	131.6 ± 27.8	0.86
Multilobar involvement (CXR)	917 (59.2%)	734 (57.0%)	183 (69.9%)	<0.01
Tachypnea^a^	313 (20.1%)	256 (19.8%)	57 (21.7%)	0.50
Tachycardia^b^	83 (5.3%)	74 (5.7%)	9 (3.4%)	0.13
Acute confusion	225 (14.5%)	178 (13.8%)	47 (18.0%)	0.08
Hypoxemia^c^	337 (21.7%)	280 (21.7%)	58 (21.7%)	0.99
Arterial pH < 7.35	858 (55.2%)	706 (54.7%)	152 (57.8%)	0.36
SMART-COP score	3 (2, 4)	2 (2, 4)	3, (2, 4)	<0.01^d^
Ventilator use	271 (17.4%)	225 (17.4%)	46 (17.5%)	0.98
ICU admission	238 (15.3%)	186 (14.4%)	52 (19.8%)	0.03
AKI	277 (17.8%)	157 (12.2%)	120 (45.3%)	<0.01
Hospital days	11 (7, 18)	10 (6, 17)	12 (8, 19)	0.01^d^
Mortality	162 (10.4%)	120 (9.3%)	42 (16.0%)	<0.01

CKD, chronic kidney disease; SBP, systolic blood pressure; CXR, chest x-ray; ICU; intensive care unit; AKI, acute kidney injury.

^a^Tachypnea defined by age-adjusted criteria: age ≦50 years, ≧25 breath/min; age >50 years, ≧30 breaths/min.

^b^Tachycardia defined by heart rate: ≧125 beats/min.

^c^Hypoxemia defined by age-adjusted criteria: age ≦50 years, oxygen saturation ≦93%; age >50 years, oxygen saturation ≦90%.

^d^Compared by Exact Wilcoxon two-sample test.

Of the 271 patients who required ventilators during hospitalization, 46 had CKD and 225 did not. Those with CKD demonstrated significantly lower blood pH values and bicarbonate and hemoglobin levels. The ventilator type; serum albumin, CRP, and arterial carbon dioxide levels; ventilator support duration; and in-hospital mortality were not significantly different between those with and those without CKD (Table 3). These findings suggest that in patients who required ventilator support, the disease severities were similar between those with and those without CKD.

**Table 3:** Characteristics of ventilator users.

Characteristics	Total	Non-CKD	CKD	p value
Number	271	225	46	N/A
Male	166 (61.3%)	133 (59.1%)	33 (71.7%)	0.11
Age (years)	79.9 ± 13.0	80.0 ± 13.1	80.0 ± 12.9	0.97
Hb (g/dL)	11.2 ± 2.3	11.4 ± 2.2	10.0 ± 2.3	<0.01
WBC (10^3^/μL)	14.0 ± 7.7	14.1 ± 7.7	13.2 ± 7.7	0.44
Albumin (g/dL)	2.8 ± 0.5	2.8 ± 0.5	2.7 ± 0.6	0.60
CRP (mg/dL)	12.0 ± 10.4	11.3 ± 9.9	14.9 ± 12.5	0.08
pH	7.38 ± 0.1	7.39 ± 0.1	7.35 ± 0.1	0.04
PaO_2_ (mmHg)	92.9 ± 71.8	94.5 ± 71.6	85.4 ± 73.1	0.44
HCO_3_^−^(mmol/L)	26.4 ± 7.8	26.5 ± 8.0	24.3 ± 6.3	0.04
PaCO_2_ (mmHg)	46.5 ± 21.3	46.4 ± 20.6	47.0 ± 24.2	0.87
Ventilator type				0.23
Invasive	149 (55.0%)	120 (53.3%)	105 (46.7%)	
Non-invasive	122 (45.0%)	29 (63.0%)	17 (37.0%)	
Ventilator days	11 (5, 28)	11 (5, 26)	11.5 (5, 30)	0.71^a^
Mortality	81 (29.9%)	65 (28.9%)	16 (34.8%)	0.43

CKD, chronic kidney disease; Hb, hemoglobin; CRP, C-reactive protein.

^a^Compared by Exact Wilcoxon two-sample test.

Of the 1554 participants, 238 were admitted to the ICU during hospitalization; 52 had CKD and 186 did not. Patients with CKD demonstrated significantly lower serum hemoglobin and bicarbonate levels and blood pH values. Sex, age, and serum albumin and CRP levels were not significantly different. In patients admitted to the ICU, the duration of ICU care and mortality were not significantly different between the patients with and those without CKD (Table 4). These findings suggest that in patients admitted to the ICU, the disease severities were similar between those with and those without CKD.

**Table 4:** Characteristics of the patients admitted to the ICU.

Characteristics	Total	Non-CKD	CKD	p value
Number	238	186	52	N/A
Male	158 (66.4%)	119 (64.0%)	39 (75.0%)	0.14
Age (years)	78.7 ± 12.9	78.7 ± 13.1	78.5 ± 12.6	0.92
WBC (10^3^/μL)	14.1 ± 7.8	14.2 ± 7.9	137 ± 7.7	0.70
Hb (g/dL)	11.2 ± 2.5	11.5 ± 2.4	10.2 ± 2.6	< 0.01
Albumin (g/dL)	2.8 ± 0.5	2.8 ± 0.5	2.7 ± 0.5	0.67
pH	7.38 ± 0.10	7.39 ± 0.10	7.35 ± 0.12	0.02
PaCO_2_ (mmHg)	43.2 ± 20.6	43.5 ± 19.8	42.2 ± 23.2	0.70
PaO_2_ (mmHg)	90.3 ± 65.1	91.3 ± 63.4	86.8 ± 70.8	0.66
HCO_3_^−^ (mmol/L)	24.2 ± 7.4	24.9 ± 7.7	21.9 ± 6.1	< 0.01
CRP (mg/dL)	13.1 ± 10.8	12.3 ± 10.1	15.4 ± 13.1	0.16
ICU days (days)	6 (3, 14)	6 (3, 15)	7 (3, 13.5)	0.64^a^
Mortality	56 (23.5%)	40 (21.5%)	16 (30.8%)	0.16

CKD, chronic kidney disease; WBC, white blood cell; Hb, hemoglobin; ICU, intensive care unit; CRP, C-reactive protein.

^a^Compared by Exact Wilcoxon two-sample test.

### Prediction capability of eGFR on the outcomes of pneumonia

**Figure 2:**
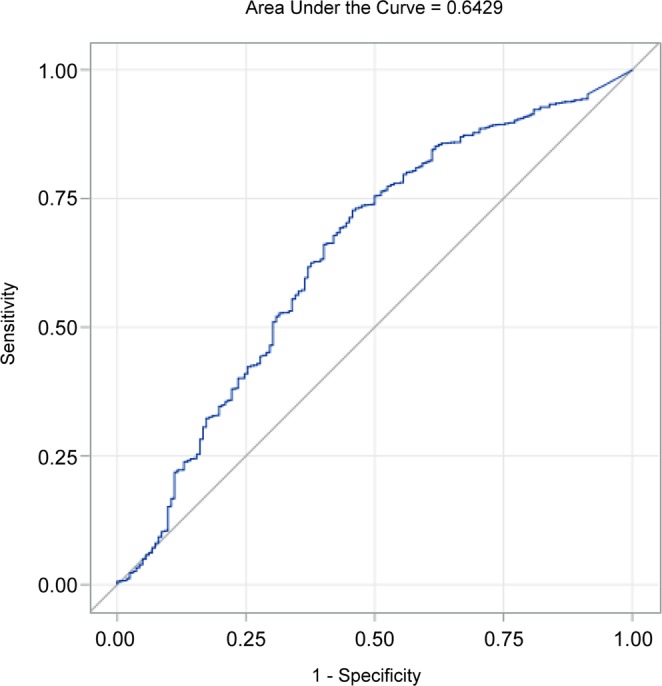
ROC curve of eGFR to predict in-hospital mortality in patients hospitalized with pneumonia. The ROC curve with Youden criteria was used to determine the optimal cutoff eGFR value in the univariate logistic regression model. The optimal cutoff value for prediction of in-hospital mortality was 56 mL/min/1.73 m^2^. The sensitivity and specificity of this cutoff value was 0.73 and 0.54, respectively, with and area under the curve of 0.64. ROC, receiver operating characteristics; eGFR, estimated glomerular filtration rate.

The ROC curve with Youden criterion of eGFR showed that the area under the curve (AUC) was 0.64 and the optimal cutoff eGFR was 56 mL/min/1.73 m^2^. This finding indicated that patients hospitalized with pneumonia with an eGFR <56 mL/min/1.73 m^2^ were at a higher risk of in-hospital mortality (Fig. 2).

**Figure 3:**
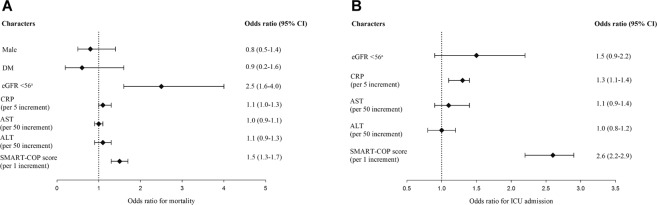
Risk for adverse outcomes of pneumonia according to multivariate logistic regression analysis. (**A**) Risk for mortality (**B**) Risk for ICU admission. CI, confidence interval; ICU, intensive care unit; DM, diabetes mellitus; eGFR, estimated glomerular filtration rate; CRP, C-reactive protein; AST, aspartate aminotransferase; ALT, alanine aminotransferase. ^a^eGFR in mL/min/1.73 cm^2^.

Logistic regression was used to determine the association of in-hospital mortality and with the cutoff eGFR value and other predictors. In the univariate logistic regression, eGFR <56 mL/min/1.73 m^2^; serum levels of CRP, aspartate transaminase, and alanine transaminase; and SMART-COP score were associated with in-hospital mortality (p ≦ 0.2, data not shown). Age was not included in the multivariate logistic regression model to avoid collinearity because it was included in the SMART-COP score. In the multivariate logistic regression, only eGFR <56 mL/min/1.73 m^2^, serum CRP level, and SMART-COP score demonstrated a significant association with the risk of mortality. Patients with eGFR <56 mL/min/1.73 m^2^ had an OR of 2.5 (95% CI 1.6–4.0) for in-hospital mortality (Fig. 3A). With a sample size of 1554, H0 of 0.1 and α error probability of 0.05, the statistical power was 0.99. Given a confidence level of 95%, sample size of 1554, population size of 2,300,000 residents of Taipei City, and a percentage of 50%, the confidence interval is 2.49%. Furthermore, we used the same cutoff eGFR value to predict ICU admission. In the univariate analysis, eGFR <56 mL/min/1.73 m^2^ along with other predictors that associated with ICU admission (p ≦ 0.2, data not shown) were included into multivariate logistic regression model. In the multivariate analysis, this cutoff eGFR value was not significantly associated with ICU admission (OR 1.5, 95% CI 0.9–2.2). Notably, higher CRP levels and SMART-COP scores were significantly associated with ICU admission in the multivariate logistic regression model (Fig. 3B). These findings suggest that eGFR <56 mL/min/1.73 m^2^ is an independent predictor of in-hospital mortality in patients hospitalized with pneumonia, which highlights the adverse effects of renal impairment on the outcome. However, this cutoff value is not a valid predictor of ICU admission.

**Figure 4:**
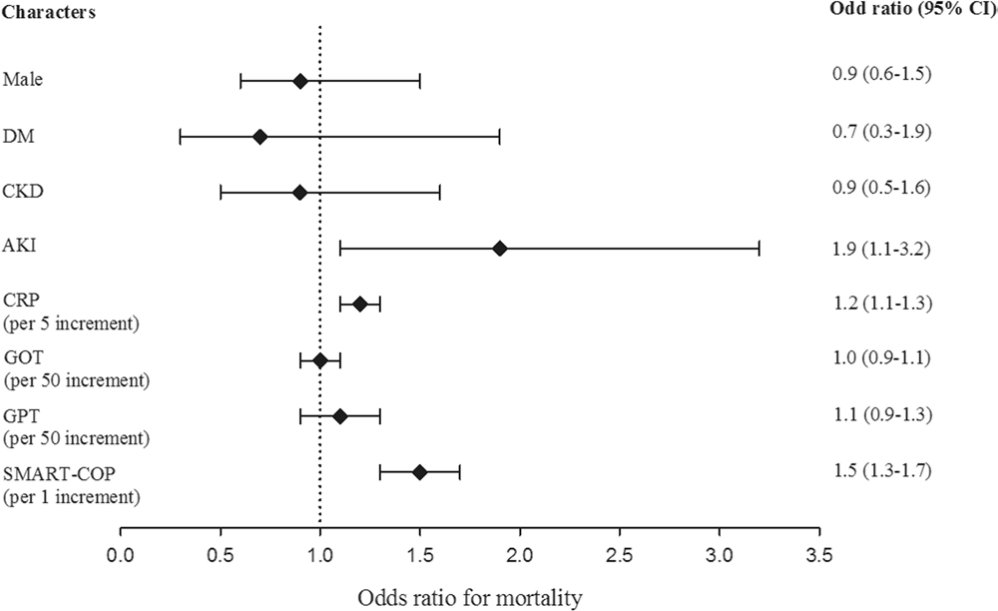
Comparative risk of CKD and AKI for mortality in patients hospitalized with pneumonia. CKD, chronic kidney disease; AKI, acute kidney injury; CI, confidence interval; DM, diabetes mellitus; CRP, C-reactive protein; AST, aspartate aminotransferase; ALT, alanine aminotransferase.

**Figure 5:**
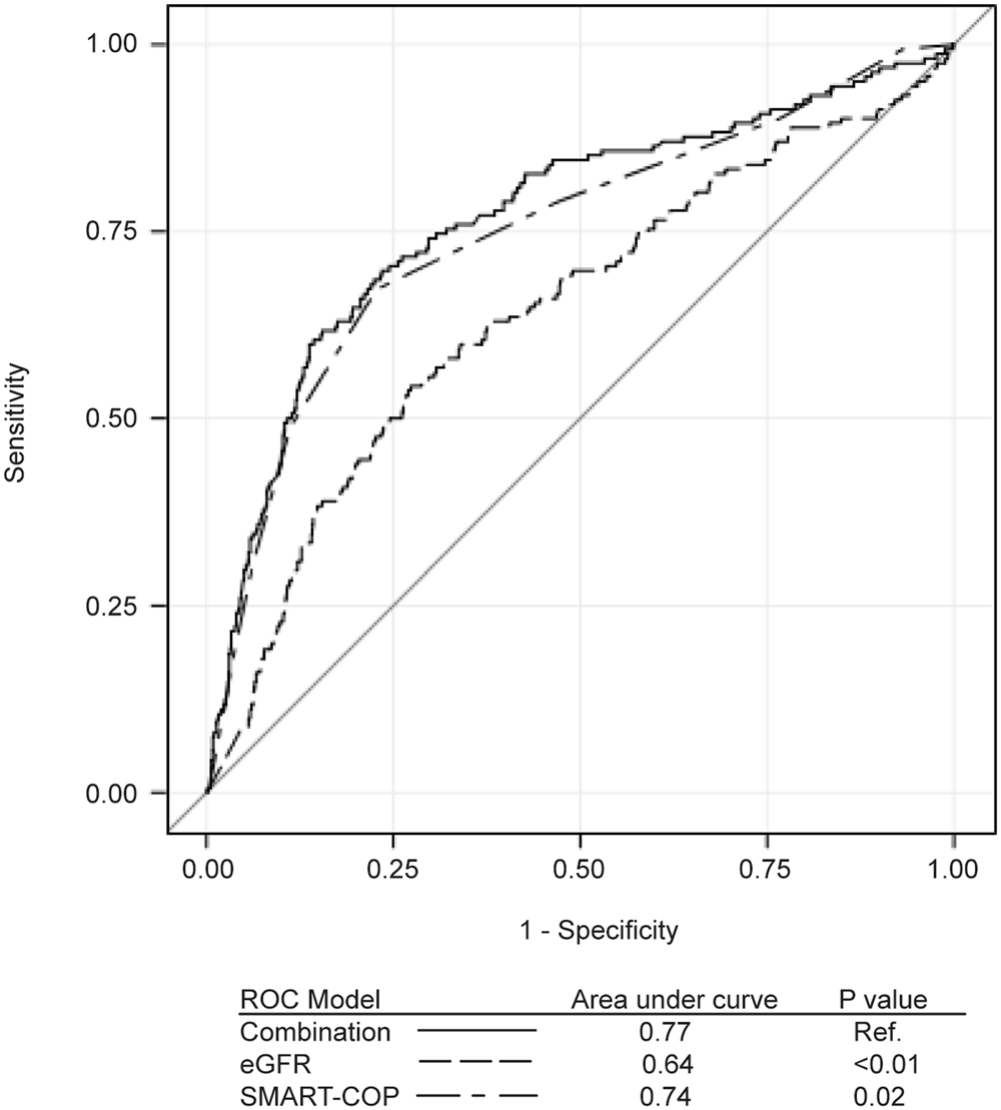
ROC curves of eGFR, SMART-COP score, and a model with the two covariates combined. ROC, receiver operating characteristics; eGFR, estimated glomerular filtration rate.

While eGFR <56 mL.min/1.73 m^2^ predicts mortality of patients hospitalized with pneumonia, decreased renal function may result from either CKD, AKI, or both. In order to define the relative contribution each factor on pneumonia-related mortality, a multivariate logistic regression model that included both CKD and AKI were used for this analysis. In this model, CKD was not significantly associated with risk of mortality. In contrast, AKI was significantly associated with risk of mortality in patients hospitalized with pneumonia, indicating a stronger contribution of AKI than CKD on pneumonia-related mortality. (Figure 4) To further confirm the eGFR value in predicting in-hospital mortality in patients with pneumonia, we compared the ROC curves of eGFR, SMART-COP score, and a model with both predictors combined. The ROC curve of the model with combined eGFR and SMART-COP score demonstrated significantly higher AUC than that of either of the predictors (Fig. 5). This indicates that the addition of eGFR improves the predictive capability of SMART-COP score for pneumonia-associated mortality and confirms the eGFR value in predicting in-hospital mortality in patients hospitalized with pneumonia.

## Discussion

The main finding of this study was that eGFR <56 mL/min/1.73 m^2^ obtained in the emergency department independently predicts in-hospital mortality in patients with pneumonia. Furthermore, the addition of eGFR to SMART-COP score improves the latter’s capability to predict in-hospital mortality. Another significant finding was that patients with CKD demonstrated higher SMART-COP scores, had pneumonia with more multilobar involvement, had higher in-hospital mortality and ICU admission rates, and had longer hospitalization. However, logistic regression model including both CKD and AKI showed that AKI contributed more than CKD on pneumonia-related mortality.

Previous studies showed that CKD is associated with an increased risk of various infectious diseases and poor outcomes secondary to infection-related hospitalization^19,20,28,29^, which could be attributed to the suboptimal immunity of patients with CKD. Numerous studies demonstrated the disturbed innate and adaptive immunities in patients with ESRD^30,31^. Furthermore, proinflammatory cytokinemia, which is associated with an increased risk of mortality, was observed in patients on hemodialysis^32^. These findings implied that decreased renal function may influence immune function and may be a predictor of outcomes in patients with pneumonia.

Two community-based studies demonstrated the association between CKD and the risk of pneumonia-related hospitalization and mortality. A retrospective cohort study with 252,516 participants in Canada reported an increased risk of pneumonia-related hospitalization and mortality in patients with decreased eGFR^22^. Another retrospective cohort study involving elderly patients with diabetes (≧65 years) reported that eGFR <30 mL/min/1.73 m^2^ at the time of diagnosis of diabetes is related to higher 28-day and 90-day mortality rates after hospitalization due to community-acquired pneumonia^24^. Based on these studies, this hospital-based study demonstrated that a single eGFR measurement in the emergency department is an independent predictor of in-hospital mortality in patients with pneumonia, which in turn provides valuable information to clinical practitioners.

Our data revealed the association between renal function and severity scores of pneumonia. CURB-65 includes blood urea nitrite^13^, which is a renal function marker, as one of its scoring factors, which could explain the significantly lower eGFR in groups with higher CURB-65 scores. By contrast, SMART-COP score does not consider renal function markers; it includes more respiratory parameters into its scoring system^14^. This explains the insignificant difference in eGFR between groups stratified by SMART-COP scores and allows the combination of SMART-COP score and eGFR in a multivariate logistic regression model to enhance the statistical strength while avoiding the effect of collinearity.

Moreover, our data suggested that decreased eGFR is not a reliable predictor of ICU admission. Decreased eGFR is not directly associated with ventilator use, and as the requirement of ventilator support is a major indication for ICU admission, decreased eGFR is not an ideal predictor of ICU admission. In contrast to our finding, the association between renal failure and respiratory disorders had been reported in a small number of studies. In patients with ESRD, fluid overload was associated with restrictive and obstructive respiratory disorders^33^. Additionally, hemodialysis-related hypoxemia due to CO_2_ diffusion into the dialysate with compensative hypoventilation was suggested^34^. A possible explanation for the discrepancy is that our study included a relatively small number of patients with ESRD. Therefore, the effect of ESRD on respiratory disorders is less significant in this study.

Furthermore, the suboptimal predictive capability of eGFR for ICU admission could be due to differences in the medical decisions between physicians^16^ and the individual considerations of each patient. For example, in a patient with terminal disease status, invasive ventilator support may not be considered even if severe pneumonia with respiratory failure is present, and the patient may be admitted to the general ward for conservative treatment. Therefore, ICU admission may be less relevant to the severity of pneumonia and, thus, affect the accuracy of the predictors.

Another finding of our study is that higher SMART-COP score is independently associated with ICU admission. As mentioned previously, respiratory failure is one of the major indications of intensive care; thus, the significant association between SMART-COP score, which includes multiple respiratory parameters, and ICU admission is reasonable. Consistent with our finding, Charles *et al*. reported that SMART-COP score is associated with the need for invasive respiratory support, thereby confirming that SMART-COP score is a potential predictor of ICU admission in patients with pneumonia^14^.

The limitations of our study include its retrospective design with some unadjusted confounding factors, including smoking status and residence environment, the unspecified causes of death, and unavailable data for PSI calculation. The strengths of our study include a relatively sufficient number of participants and completely available medical records.

In conclusion, eGFR <56 mL/min/1.73 m^2^ is an independent predictor of in-hospital mortality in patients hospitalized with pneumonia, indicating that even mild renal impairment at emergency department affects the outcome of pneumonia adversely. The result of this study infers that in patients with decreased renal function, additional care should be provided to reduce pneumonia-related mortality. The guidelines for infection control and treatment in patients with decreased renal function are still lacking; thus, further research is warranted in this field.
